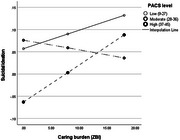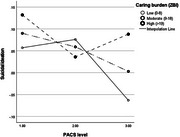# Positive experience of caregiving moderates suicidal ideation in family caregivers of community‐dwelling older adults with neurocognitive disorders

**DOI:** 10.1002/alz70860_102978

**Published:** 2025-12-23

**Authors:** Zhaohua Huo, Benjamin HK Yip, Allen Lee, Sheung Tak Cheng, Wai Chi Chan, Ada WT Fung, Suk Ling Ma, Calvin PW Cheng, Frank HY Lai, Samuel YS Wong, Linda Lam

**Affiliations:** ^1^ The Chinese University of Hong Kong, New Territories, Hong Kong SAR, China; ^2^ The Education University of Hong Kong, New Territories, Hong Kong SAR, China; ^3^ The University of Hong Kong, Hong Kong Island, Hong Kong SAR, China; ^4^ Hong Kong Baptist University, Kowloon, Hong Kong SAR, China; ^5^ Northumbria University, Newcastle, United Kingdom

## Abstract

**Background:**

Psychological distress is common in family carers of persons with neurocognitive disorders (NCDs). This study evaluated suicidal ideation in family caregivers of persons with NCDs, and the roles of positive experience of caregiving in moderating these severe psychological difficulties.

**Method:**

445 older adults (NCDs: 322, normal cognition: 123) and their family caregivers were recruited from the population‐based Hong Kong Mental Morbidity Survey for Older Persons. Carers’ suicidal ideation was screened by positive response from Patient Health Questionnaire‐9 (PHQ‐9): thoughts being better off dead or hurting yourself in the past two weeks. Positive aspects of caregiver (PAC) were measured. A conceptual model was constructed to test the mediation and moderation effects among PAC, carer burden, psychological distress and suicidal ideation.

**Result:**

Nearly one in eleven (9%) dementia caregivers reported thoughts of death or suicidal ideation in the past two weeks. Higher rates were found in female carers comorbid with mood disorders and carers of high comorbidity or dependence persons. The association between carer burden and suicidal ideation was mediated by psychological distress (80.5%, *p* = 0.023). Higher levels of PAC were associated with lower rates of suicidal ideation in carers with moderate‐to‐high caring burden (moderation effects of PAC, *p* <0.05).

**Conclusion:**

Suicidal ideation in carers was associated with caring burden and affected by psychological distress. PAC attenuates the impact of burden and distress on suicidal ideation. Carer psychological intervention should focus on strengthening PAC, especially in carers experiencing high psychological distress.